# Ameliorating Effect on Aβ-Induced Alzheimer’s Mice by *Litsea cubeba* Persoon Powder

**DOI:** 10.3390/molecules26185709

**Published:** 2021-09-21

**Authors:** Kuan-Tseng Lee, Chen-Yeon Chu, Shen-Shih Chiang

**Affiliations:** 1 Department of Food Science and Biotechnology, National Chung Hsing University, 145 Xingda Rd., South Dist., Taichung 40227, Taiwan; jason51217@hotmail.com; 2 Institute of Green Products, Feng Chia University, Taichung 40724, Taiwan; cychu@mail.fcu.edu.tw

**Keywords:** Alzheimer’s disease, amyloid β protein, *Litsea cubeba* Persoon, neurotoxicity, cognitive impairment

## Abstract

Alzheimer’s disease (AD) is caused by excessive oxidative damage and aging. The objective of this study was to investigate the anti-dementia effect of LCP fruit powder on amyloid β (Aβ)-induced Alzheimer’s mice. The composition of LCP essential oil was determined by gas chromatography/mass spectrometry. In addition, the water maze was used to evaluate the learning and memorizing abilities of the mice. The concentrations of malondialdehyde (MDA), protein carbonyl, phosphorylated τ-protein, and the deposition of Aβ plaques in mouse brains were also assessed. The results showed that the main components of essential oils in LCP and d-limonene, neral, and geranial contents were 14.15%, 30.94%, and 31.74%, respectively. Furthermore, oral administration with different dosages of LCP significantly decreased the escape time (21.25~33.62 s) and distance (3.23~5.07 m) in the reference memory test, and increased the duration time (26.14~28.90 s) and crossing frequency (7.00~7.88 times) in the target zone of probe test (*p* < 0.05). LCP also inhibited the contents of MDA and the phosphor-τ-protein from oxidative stress, reduced the brain atrophy by about 3~8%, and decreased the percentage of Aβ plaques from 0.44 to 0.05%. Finally, it was observed that the minimum dosage of LCP fruit powder (LLCP, 30.2 mg/day) could prevent oxidative stress induced by Aβ and subsequently facilitate memory and learning deficits in Aβ-induced neurotoxicity and cognitively impaired mice.

## 1. Introduction

Population aging has become a common social phenomenon in the 21st century, and it has a severe impact on the political, economic, and social aspects of various countries [1].

Senile dementia is a neurodegenerative disease caused by aging and excessive oxidative damage in the body. Among the types of dementia, Alzheimer’s disease (AD) is the most prevalent and has caused the medical burden of the aging society in various countries to increase accordingly [2,3]. AD is a progressive neurodegenerative disorder caused by amyloid β (Aβ) plaque deposition, and tau protein hyperphosphorylation (Phosphorylated tau protein, p-tau) causes nerve fibers to entangle in the hippocampal gyrus, leading to a loss of memory, learning ability and cognitive dysfunction [4].

Typically, AD is classified into three stages. In the early stage (the initial 1 to 2 years), the patients have speech impediments, significant memory loss (particularly short-term memory loss), and become inactive and unmotivated. In the middle stage (2 to 5 years), the patients can no longer live alone as they often cannot cook, clean, or shop and may hallucinate. Finally, in the latest stage (more than five years), patients are incapable of communicating and walking, have bladder and bowel incontinence, and are confined to a wheelchair or bed, followed by death [5,6,7,8].

The Neurofibrillary tangles (NFT) observed in AD patients are the collections of paired helical filaments which are composed of hyperphosphorylated tau. Hyperphosphorylation results in dissociation, destabilization of the microtubules, and the oligomerization of the tau proteins. The NFT can spread from neuron to neuron, causing the neuronal apoptosis and further pathogenesis of the disease. [9] Protein carbonyls are often used as the indicator to assess cellular protein oxidation, especially in protein backbone and amino acid residues (proline, arginine, lysine, threonine, and others). Moreover, they may be the secondary metabolites of amino acids (cysteine, histidine, and lysine) with reactive carbonyl compounds (ketones, aldehydes) during lipid peroxidation or glycation/glycoxidation reactions in Alzheimer’s brains [10,11].

*Listea cubeba* (Lour.) Persoon (LCP) is a perennial dioecious plant of the Lauraceae family. In China, it is known as Shan Cang Zi or shan jiao ji, while in Taiwan, it is named by the indigenous people as makauy. The plant’s fruits are spherical and approximately 4–6 mm in diameter. They are usually used in food, spices, cosmetics, flavor enhancers, and insect repellents. In ancient times, Taiwanese aborigines used the essential oil of LCP fruit to treat headaches, inflammation, intoxication, bronchitis, and dyspepsia. The dominant components of the essential oil of *L. cubeba* are monoterpenes [12]. The methanol extract fractions of LCP show remarkable antioxidant, anti-inflammatory activities and inhibit the growth of hepatocyte carcinoma cells [13,14]. The major component of the oil is the citral, and it exhibits several effects on the central nervous system, including reducing pain and anxiety, and increasing learning, memory, attention, arousal, relaxation, sedation, and sleep effects [15].

Melatonin (N-Acetyl-5-methoxytryptamine, MEL) is an endogenous neurosecretory hormone, synthesized mainly from L-tryptophan to produce serotonin and then converted to melatonin in the pineal gland [16]. It is a potent antioxidant and reduces reactive oxygen species in the brain of Alzheimer’s disease patients, which can scavenge the hydroxyl radicals and prevent the amyloid-like protein aggregation, DNA mitochondrial damage and regulate the brain oxidative stress [17,18,19,20]. Melatonin protects neurons and glia, which acts against the action of oxygen radicals generated by homocysteine. It also reduces the cells’ structural alterations and leads to a diminished contractility of neuronal degeneration [21]. The melatonin is the medical drug for Alzheimer’s disease patients in Taiwan, and belongs to (NMDA) receptor antagonists. This drug can inhibit nerve cell damage caused by the excessive effect of glutamine on the NMDA receptor, and alleviate the symptoms for moderate-to-severe Alzheimer’s disease [22]. Thus, melatonin is a potentially useful agent in deferring the pathology of AD. Soluble β-amyloid protein fragments reported in neurotoxicity are fragments 25–35, 1–28, and 1–40 secreted from cells constituent of plasma and cerebrospinal fluid [23].

Therefore, the purpose of this study aimed to examine the effect of LCP fruit powder on several behavioral tests and determine the effects on oxidative stress and pathogenicity in Aβ-induced Alzheimer’s mice.

## 2. Methods and Materials

### 2.1. Preparation and Evaluation of L. cubeba Persoon Fruit Essential Oil

The LCP fruits were procured from the Qingliu tribe, Nantou, Taiwan, on 7 August 2016. LCP fruits were dried by hot air at 50 °C for 60 h then grounded using a high-speed grinder. The powder was filtered through a 60-mesh screen and then stored at 4 °C. Proximate compositions of LCP fruit powder, including moisture, crude ash, crude fat, carbohydrate content, and crude protein, were determined according to the methods of the AOAC [24]. The nitrogen factor used for the simple protein calculation was 6.25. The carbohydrate content was calculated by subtracting crude ash, fat, fiber, and protein content and then expressed as milligrams per gram of dry weight.

Five hundred grams of fresh fruit from LCP were mixed with 1500 mL distilled water. A Clevenger-type apparatus was used to extract essential oil compounds for 6 h. The essential oils were dried by anhydrous sodium sulfate and stored at 4 °C. The compositions of essential oils were analyzed by gas chromatography (GC) and GC mass spectrometry (GC/MS).

The volatile compounds of essential oil were identified by GC/MS using a Shimadzu TQ-8040 (Kyoto, Japan) equipped with a DB-1 capillary column (60 m × 0.25 mm, ID; 0.25 μm film thickness, J&W Scientific, Folsom, CA, USA). Helium (flow rate, 0.8 mL/min) was used as the carrier gas, and injection volumes were 1 μL. The column temperature was programmed from 60 °C to 240 °C at a rate of 4 °C/min and then held constant at 240 °C for 15 min. The injection port and transfer line temperatures were 250 °C and 265 °C, respectively. The electron impact (EI) energy was 70 eV, and the ion source temperature was set at 230 °C. Electron impact mass spectra were recorded in the m/z 30–400. The obtained mass spectra were compared with the NIST14 Mass Spectral database (Scientific Instrument Services, Ringoes, NJ, USA).

### 2.2. Animals and Intra-Cerebroventricular Aβ_1–40_ Induced Surgery Model

Forty-eight male C57BL/6J mice (16 weeks old) weighing 27–29 g were purchased from National Laboratory Animal Center (Taipei, Taiwan). The mice were housed in polycarbonate cages in a controlled room, at 23 ± 2 °C, relative humidity at 60 ± 10%, and 12 h light/dark cycles. Mice were provided with rodent chow (Laboratory Rodent Diet, 5001, Labdiet PMI^®^ Nutrition International, Brentwood, MO, USA) and water during the experimental period. Bodyweight and food intake were recorded once per week.

Amyloid β-peptide_1–40_ was purchased from Sigma Chemical Co. (St. Louis, MO, USA) and subsequently dissolved in phosphate-buffered saline (PBS; 200 pmol in 5 µL) [23]. The Aβ mouse model was generated via intra-cerebroventricular injection of Aβ_1–40_ using a similar method in the previous study [25]. Mice were anesthetized with isoflurane with a flow rate of 3 L/min. Mice heads were fastened to the stereotaxic frame (DST-1, Tansheng Co., Taipei, Taiwan), and the scalps were cut. The location of the bregma was identified using the atlas of Franklin and Paxions [26]. Injection coordinates in the X-Y axis were ±1.8 mm vs.−2.3 mm bilaterally for two hippocampal cornu ammonis 1 (CA1) sites. The mini pump was filled with Aβ_1–40_ solution and connected to a 26-gauge needle micro-injector syringe for infusion with 0.5 µL/min Aβ_1–40_ solutions (400 pmol in 5 µL) for 5 mins at 1.5 mm depth in hippocampus site. The mouse scalp was stitched up via suture needles, and mice recovered within 5 min.

### 2.3. Administration Dosages

**Figure 1:**
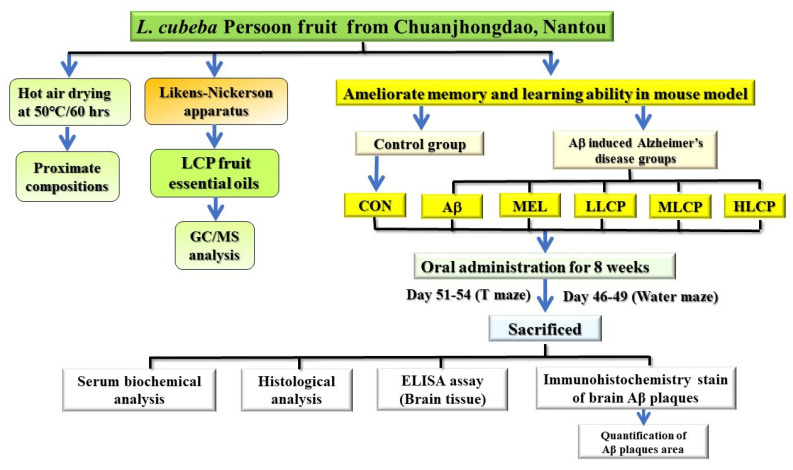
The conceptual framework of experiments for ameliorate memory and learning ability of LCP powder in the mouse model.

The active essential oil composition in *Listea cubeba* Persoon fruit powder consisted of about 10% from Table 1. Therefore, the essential oil could not feed directly to the mice. Generally, the essential oil was diluted to 1% for commercial use. Consequently, the powder was used to replace the pure essential oil, based on the active dose fed by Chen et al. [27] and the yield of essential oils in this study. The LCP powder with the same essential oil content was used as the feeding sample for subsequent tests. The oral LD_50_ of LCP essential oil was 3690 mg/kg (close to 4000 mg/kg, the 95% confidence interval range is 2710–5010 mg/kg), which was implemented by the acute toxicity class method according to the initial dose prescribed by the Globally Harmonized Classification System (GHS) in Luo et al. [28]. In this study, the highest dosage essential oil of LCP was 500 mg/kg which was far less than the LD_50_ of the LCP essential oil (3690 mg/kg). Therefore, the mice were divided into six groups; each group had eight mice and designations: Group (1), orally administered with water (CON). Group (2), Aβ_1–40_ induced group and orally administered with water (Aβ). Group (3), Aβ_1–40_ induced and orally administered with 0.29 mg/day melatonin (Sigma-Aldrich Inc., Michigan, USA) per day (MEL). Group (4), Aβ_1–40_ induced and orally administered with low dosage (30.2 mg/day) of LCP fruit powder (LLCP); Group (5), Aβ_1–40_ generated and orally administered with medium dosage (90.6 mg/day) of LCP fruit powder (MLCP). Group (6), Aβ_1–40_ induced and orally administered with high dosage (151 mg/day) of LCP fruit powder (HLCP). The sample is mixed in sterile water and administered directly by tube feeding. All mice were orally administered with the samples by daily gavage for eight weeks. The experimental procedures were reviewed and approved by the Institutional Animal Care and Use Committee (IACUC No. 104-087) of National Chung-Hsing University. The scheme describing the treatments and protocols of LCP powder in this study is shown in Figure 1.

### 2.4. Water Maze Test

According to previously established methods, the water maze test was performed on days 46 to 49 following surgery [25] to assess mice’s learning and memory ability impairments. The water maze apparatus was a plastic circular tank (125 cm in diameter and 45 cm in height) with a movable stainless steel platform (12 cm in diameter and 25 cm height) in the first quadrant. The apparatus was filled with water (27 cm, 25 °C) and made opaque to hide the platform during the test. Trials recorded using a video tracking system (Etho vision XT 4, Noldus Co, Leesburg, FL, USA) divided the tank apparatus into four quadrants. The movement path for each trial was monitored using the video tracking system.

### 2.5. Reference Memory Test

The reference memory test started on days 46 to 48. Each mouse received four trials per day, and each trial consisted of 90 sec. The escape platform was set in quadrant (I) during the test, and the mouse was placed into the quadrant (III). The swimming time and distance to find the escape platform was recorded. If the mouse could not find the escape platform in 90 s, the test was terminated and the mouse was guided to the escape platform, kept on the platform to rest for 30 s, and the subsequent trial was started [29].

### 2.6. Probe Test

Probe tests were started on day 49. Before the test, the escape platform was removed from quadrant I of the water maze. During the trial, each mouse had one chance for 90 s to swim in the water maze. The 90 s swimming pathway, crossing time, and frequency in the target zone were recorded by a camera with a video tracking system [29].

### 2.7. T-Maze Test

T-maze test was performed according to the method of Deacon and Rawlins [30] with some modifications. This test was designed to assess mice’s learning and memory ability impairments from days 51 to 54. The T-maze apparatus was made of black acrylic sheets. The length, width, and height were 70, 10, 20 cm, respectively. Before the test, each mouse was placed on a restricted diet for 10 h to increase the motivation to eat. On day 51, cheese was placed at the end of the left and right arms and the mouse was placed on the start arm of the T-maze. Each mouse was allowed to freely explore the T-maze for 5 min during each session. On day 52, the mouse was allowed to explore the right and left arms of the T-maze, the mouse was placed into the start arm and directed to walk into the left and right arms three times. On day 53, we defined the left arm as the correct path and the right arm as the wrong path. Thus, each mouse was forced to walk into the left and right arms three times separately. If the mouse walked into the correct path, it could eat the cheese. However, if the mouse walked in the wrong direction, it would squeeze its body and punish it in a small space for 10 s. Formal testing was performed on day 54. Each mouse had five chances for testing in the T-maze, and the percentage of correct responses was recorded. After each trial, the T-maze apparatus was wiped with 75% ethanol.

### 2.8. Sacrifice and Tissue Collection

Each mouse was fasted for 12 h and sacrificed by CO_2_ exposure. Whole blood was collected from the posterior vena cava, clotted at room temperature for 2 h, then centrifuged (3000× *g* for 20 min at 4 °C) to separate the plasma from serum. The serum was stored at −80 °C in a refrigerator until analysis. Next, the heart, liver, kidney, spleen, testis, epididymis, and brain were removed and weighed. Hepatic lobes, kidneys, and half of the brain were immersed in 10% neutral formalin and allowed to fix for 2 weeks. After fixation, the samples were rinsed in water and immersed in different ethanol concentrations for 15 h. After that, the pieces were kept for 1 h in an absolute ethanol–xylene mixture, 4 h in xylene, and embedded in warm paraffin wax for 6 h. Finally, the samples were sectioned at 6 μm and stained with hematoxylin-eosin for histological analysis. The other parts of the brain were stored at −80 °C. They identified the concentration of malondialdehyde (MDA), protein carbonyl, phosphorylated τ-protein, and quantified the depositions of Aβ plaques in the brain using immunohistochemistry.

### 2.9. Analyses of Serum Biochemical Parameters

Shang Jie Clinical Laboratory analyzed biochemical serum parameters, such as the alanine aminotransferase (ALT) (Roche Cobas #11876805, Indianapolis, IN, USA), aspartate aminotransferase (AST) (Roche Cobas #11876848), blood urea nitrogen (BUN) (Roche Cobas #11729691), creatinine (Roche Cobas #11875418), uric acid (Roche Cobas #11875426), total cholesterol (TC) (Roche Cobas #11491458), high-density lipoprotein cholesterol (HDL-C) (Roche Cobas #04713257), and low-density lipoprotein cholesterol (LDL-C) (Roche Cobas #04714423).

### 2.10. Determination of MDA Content in the Brain

Brain tissues were homogenized with ten strokes of a homogenizer in phosphate-buffered saline (PBS). The homogenate was centrifuged at 1600× *g* for 10 min at 4 °C, and the supernatant was used to assay the concentration of MDA at 530 nm using the Cayman thiobarbituric acid reactive substances (TBARS) assay kit (Cat. No. 10009055, Ann Arbor, MI, USA).

### 2.11. Determination of Protein Carbonyl and Phosphorylated τ-Protein Contents in Brain

The homogenate was centrifuged at 10,000× *g* for 15 min at 4 °C, and the supernatant was used to assay the protein carbonyl concentration at 360–385 nm by Cayman protein carbonyl colorimetric assay kit (Cat. No. 10005020, Ann Arbor).

The homogenate was centrifuged at 3000× *g* for 10 min at 4 °C, and the supernatant was used to assay the concentration of phosphorylated τ-protein at 450 nm using a phosphorylated τ-protein assay kit (P&C Biotech, Inc., Cat. No. E93893M, Miaoli, Taiwan).

### 2.12. Immunohistochemical Staining of Aβ_1–40_ in the Brain

Deparaffinization of the brain tissue was performed by submerging slides in xylene (JT Baker Co., Center Valley, PA, USA) for 40 min and then rehydrating them by immersing them in high-to-low concentrations of ethanol (95%, 90%, and 75%) each for 5 min. They were subsequently soaked in tris-EDTA buffer, and heat-induced epitope retrieval was performed for 20 min to break the protein crosslinks. The tissue slides were treated with three drops of 5% H_2_O_2_ for 5 minutes to suppress endogenous peroxidase activity, then rinsed with cold PBS. Slides were blocked with 5% goat serum and treated for 30 min to eliminate non-specific hydrophobic interactions between primary antibodies and the tissue slides. The tissue slides were incubated with three drops of primary Aβ_1–40_ antibody (Biolegend Co., San Diego, CA, USA) in a moisturizer box for 2 h, followed by three drops of secondary antibody (1–3 mg/mL, 4G8, anti-Aβ17–24 epitope; Signet, Dedham, MA, USA) and 3,3-diaminobenzidine tetra-hydrochloride (DAB) chromogen solution (Biolegend Co.). Finally, the slides were stained by hematoxylin (Muto Pure Chemical Co., Tokyo, Japan) for 30 s and reactions terminated by PBS washing. An intermittent microscope was used to observe Aβ_1–40_ plaque accumulation in the brain tissue slides. The Aβ_1–40_ plaques in brain tissue slides were quantified using ImageJ software (pro plus 6.3, Media Cybernetics Inc., Rockville, MD, USA). The percentage of Aβ_1–__40_ plaques was expressed as the percentage of total Aβ_1–40_ plaques area in brain/total brain area ×100%.

### 2.13. Statistical Analysis

The experimental data were expressed as mean ± standard deviation (SD). The data were analyzed using one-way analysis of variance (ANOVA) and Duncan’s test using a software package for the social science (SPSS), software version 20 (IBM Corp., Armonk, NY, USA). Data were considered statistically significant if *p* < 0.05.

## 3. Results

### 3.1. The Proximate Analyses and Essential Oil Content of LCP Fruits

The LCP fruits were collected on August 7, 2016, with an elevation height of 400–500 m in Qingliu tribe, Nantou, Taiwan. Table 1 shows the proximate compositions and essential oil contents of LCP fruits. The contents of moisture, crude ash, crude protein, crude fat, carbohydrate, and essential oil recovery were 65.26 ± 0.04, 1.10 ± 0.08, 4.37 ± 0.10, 10.18 ± 0.27, 19.07 ± 0.23%, and 3.80 ± 0.13, respectively.

**Figure 2:**
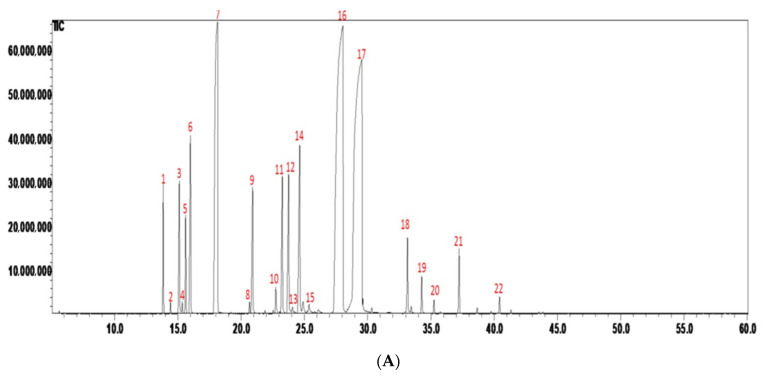

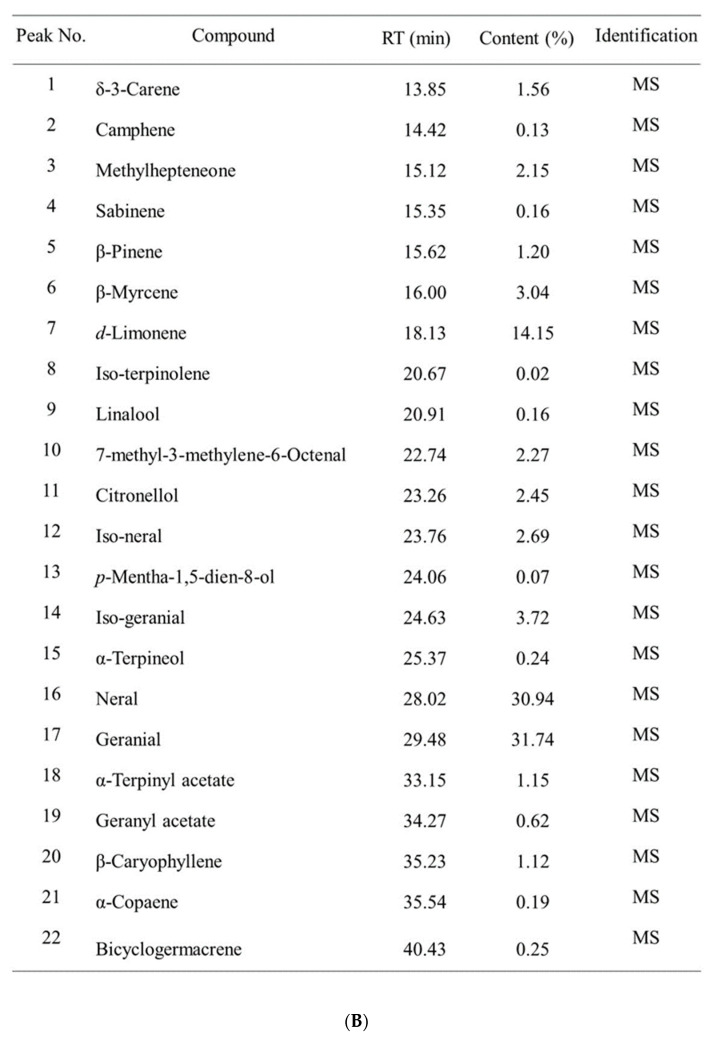
(**A**) GC chromatograms and (**B**) GC/MS analysis of volatile compounds of Litsea cubeba Persoon fruit essential oil from Alang Gluban tribe of Nantou County in Taiwan. Mass spectra compared with those of the NIST14 Mass Spectral database.

The Likens-Nickerson apparatus extracted the LCP fruits that yielded 35.92 ± 1.60 mL/kg essential oil. Figure 2A shows the GC chromatograms of *Litsea cubeba* Persoon fruit essential oil from the Alang Gluban tribe of Nantou County in Taiwan. Figure 2B demonstrates the results of crucial oil volatile compounds from fresh LCP fruit in Figure 2A. Twenty-two volatile compounds were identified and quantified from the essential oil. They mainly consisted of monoterpenes (22.41%) and oxygenated monoterpenes (74.28%). The main monoterpene constituents were *d*-limonene (14.15%), β-myrcene (3.04%), and methylhepteneone (2.15%). The main oxygenated monoterpenes constituents were geranial (31.74%) and neral (30.94%), which composed around 80% of the essential oil.

The Likens-Nickerson apparatus was used to extract essential oils from the LCP fruit. The GC/MS analysis determined the main volatile compounds of LCP fruit essential oil to be *d*-limonene (14.15%) and citral (geranial + neral) (62.68%); see many previous reports and this article’s results [28,31,32,33]. The LCP fruit essential oils were composed of monoterpenes and sesquiterpenes. The main volatile compounds were d-limonene, geranial, and neral.

### 3.2. Effects on Body Weight, Food Intake, and Relative Organ Weight

Table S1 shows the effect of *L. cubeba* Persoon fruit powder on body weight and food intake in Aβ-induced Alzheimer’s mice. Before being fed with the LCP powder, mice’s body weights in all groups were between 28.22~29.76 g and showed no significant difference. However, after 8 weeks of feeding, the body weight and food intake of the HLCP group (28.72 ± 1.30; 0.18 ± 0.02 g) decreased significantly compared with that of CON (31.28 ± 1.48; 0.21 ± 0.01 g) and Aβ (30.07 ± 2.15; 0.21 ± 0.02 g) groups. This may be because of the high content of essential oil in HLCP powder, which led to a more pungent smell and reduced food intake and body weight.

Table S2 reveals the result of the relative organ weight in Aβ-induced Alzheimer’s mice. The relative organ weight of the brain in the Aβ group (1.47 ± 0.10%) was not significantly different to the CON, MEL, LLCP, and MLCP groups (1.49 ± 0.05, 1.54 ± 0.11, 1.53 ± 0.11, and 1.55 ± 0.08%, respectively), except for the brain weight of the HLCP group, which was highest at 1.61 ± 0.08%. However, there were no group differences in each group’s relative heart, liver, kidney, and spleen organ weight.

### 3.3. Effects on Serum Biochemical Values

Results of serum biochemical values in Aβ-induced Alzheimer’s mice are described in Table S3. The liver and renal function parameters were the AST, ALT, BUN, creatinine, and uric acid, not significantly different in all groups. However, the concentration of TC in the HLCP group (108.25 ± 6.06 mg/dL) and levels of LDL-C in the MLCP and HLCP group (16.75 ± 3.19 and 14.75 ± 2.60 mg/dL, respectively) were intensely lower than that in the CON group. The HDL-C/LDL-C ratio in the MLCP and HLCP groups (5.59 ± 1.01 and 5.71 ± 0.99, respectively) was significantly higher than in the CON group.

### 3.4. Effects on Escape Time and of Reference Memory Task

The effects of LCP on the escape time of the water maze test for each group from the first day to the third day are shown in Table 2. On the first day, each group was familiarized with the position of the escape platform in the water maze apparatus; the time for each group to find the escape platform was between 74.31–84.31 s and showed no significant differences. On the second day, the mice in the Aβ group spent significantly more time and swam farther to find the escape platform than the other groups. On the third day, the time for the Aβ group was slightly reduced to 77.56 ± 14.62 s and there was no significant difference compared to the previous two days, while the time of the CON, MEL, and different doses of LCP powder groups (17.34 ± 10.07, 20.06 ± 9.85, 21.25 ± 10.61, 20.09 ± 7.70, and 33.62 ± 11.64 s) were significantly lower than that of the Aβ group (*p* < 0.05).

The effects of LCP on the escape distance of the water maze test for each group from the first day to the third day are shown in Table 2. In the water maze memory test, the value for finding the platform was lower, and the swimming distance value was shorter. On the first day, the total swimming distance to find the platform of each group had no significant difference from 9.39 to 10.74 m. On the second day, mice were familiar with the platform’s location and reduced the swimming distance from 5.31 to 8.67 m, which was significantly lower than that of the Aβ group (10.71 m). The swimming distance for the Aβ group on the third day was 8.24 m, which was considerably higher than that of the CON, MEL, LLCP, MLCP, and HLCP groups from 3.00 to 5.07 m (*p* < 0.05).

### 3.5. Effects on Swimming Path/Time and Crossing Frequency

**Figure 3:**
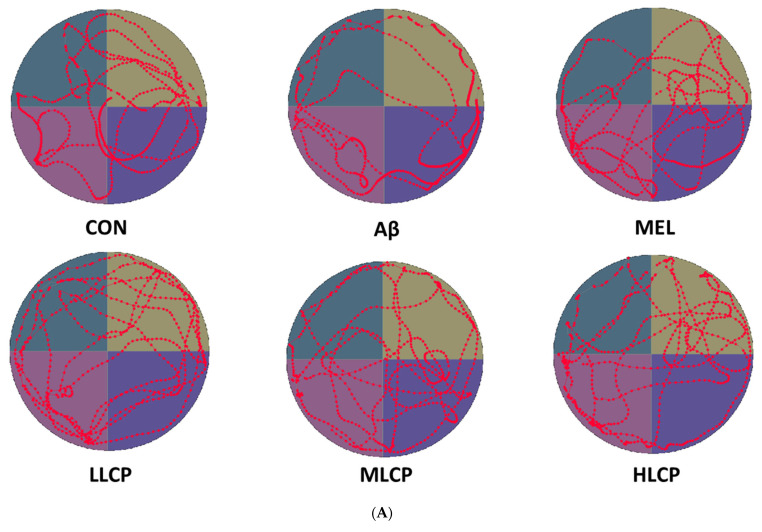

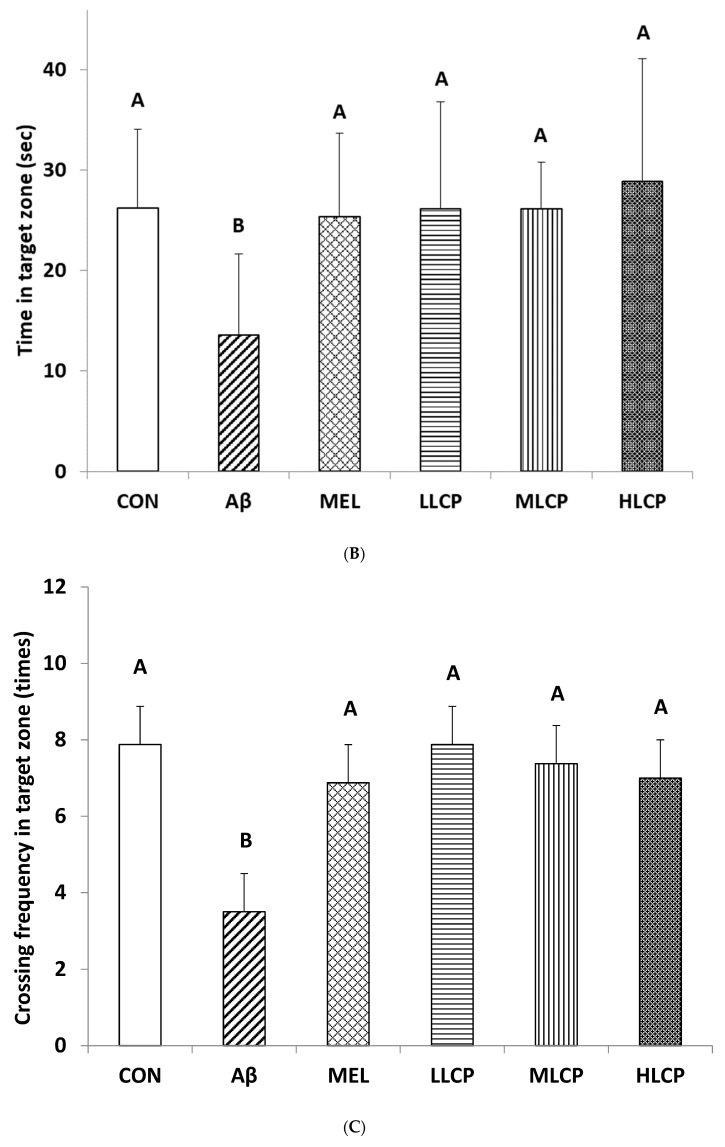

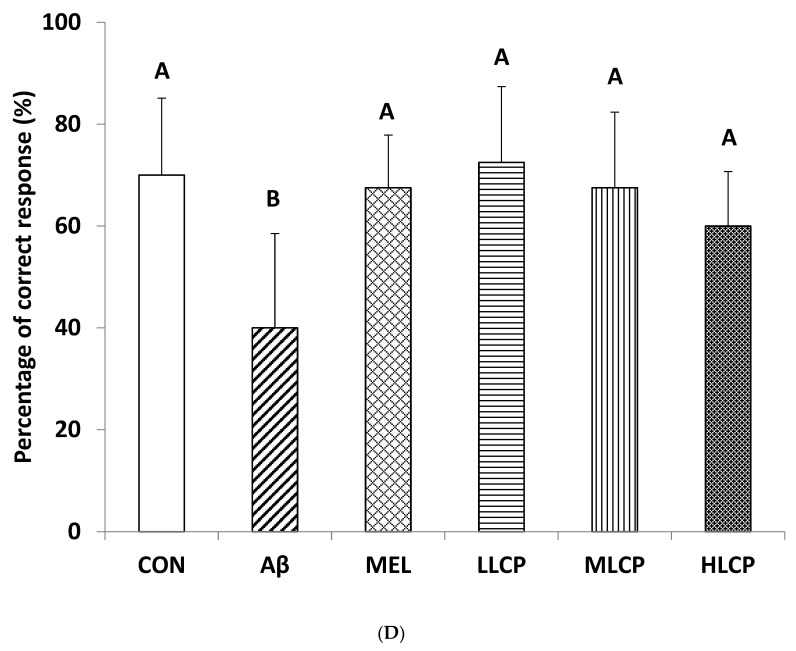
Effects of LCP fruit powder on behavior test in Aβ-induced Alzheimer’s mice. (**A**) The swimming path of probe task for each group. (**B**) Average swimming time (sec.) in target zone during probe task. (**C**) Average crossing frequency (times) in target zone during probe task. (**D**) The correct percentage of the path on the T maze discrimination task for each group. Values are expressed as mean ± SD (*n* = 8). Means with different letters (A and B) are significant differences between the groups (*p* < 0.05). Abbreviations: symbols are represented in Table 2.

Figure 3A shows the average swimming path during the 90 s of each group recorded by the video tracking system. Except for the Aβ group, the other groups swam faster to arrive at the platform zone (*p* < 0.05). The time spent and crossing frequency in the platform zone are shown in Figure 3B,C. It was found that the time spent and crossing frequency in the target zone of the Aβ group were 13.57 ± 8.09 s and 3.50 ± 2.97, respectively. These values were lower than that of MEL, LLCP, MLCP, and HLCP groups (25.39 ± 8.32, 26.16 ± 10.65, 26.15 ± 4.64 and 28.90 ± 12.20 s and 6.87 ± 1.88, 7.87 ± 1.55, 7.37 ± 1.68 and 7.00 ± 2.13 times, respectively (*p* < 0.05).

### 3.6. Effects on T-Maze Discrimination Task

In this study, the effects of LCP on learning and memory impairment were also conducted by the T-maze task. This task used high-intensity punishment and incentives to motivate the mice to choose the correct path. Figure 3D shows the percentage of mice that chose the right direction within each group. In contrast to the proper ratio of the Aβ group (40.00 ± 18.51%), mice orally administered with MEL, LLCP, MLCP, and the HLCP groups had a significantly higher probability of choosing the correct path (70.00 ± 15.11%, 72.50 ± 14.88%, 67.50 ± 14.88% and 62.50 ± 12.81%, respectively. *p* < 0.05).

### 3.7. Effects on the Concentration of Lipid Peroxidation Products, Protein Carbonyl, and Phosphorylated τ-Protein

**Figure 4:**
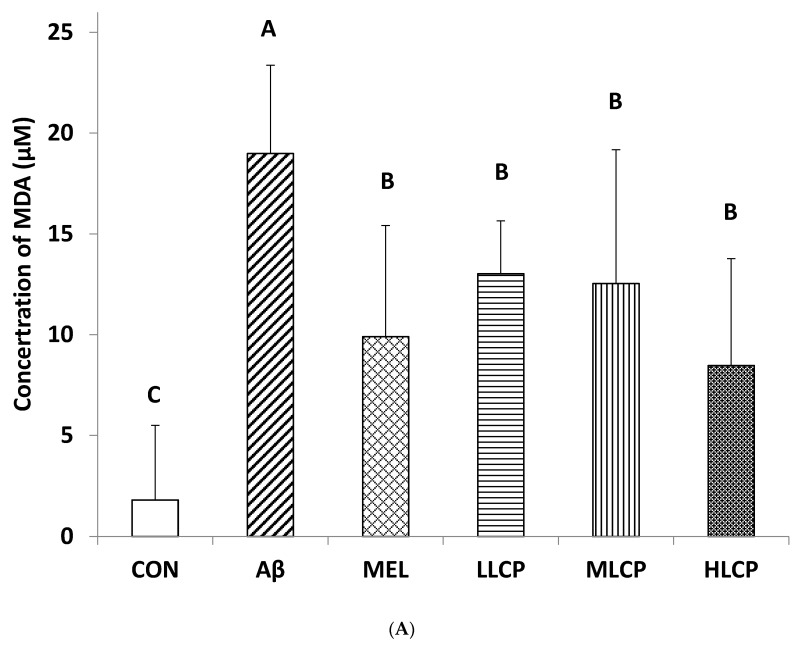

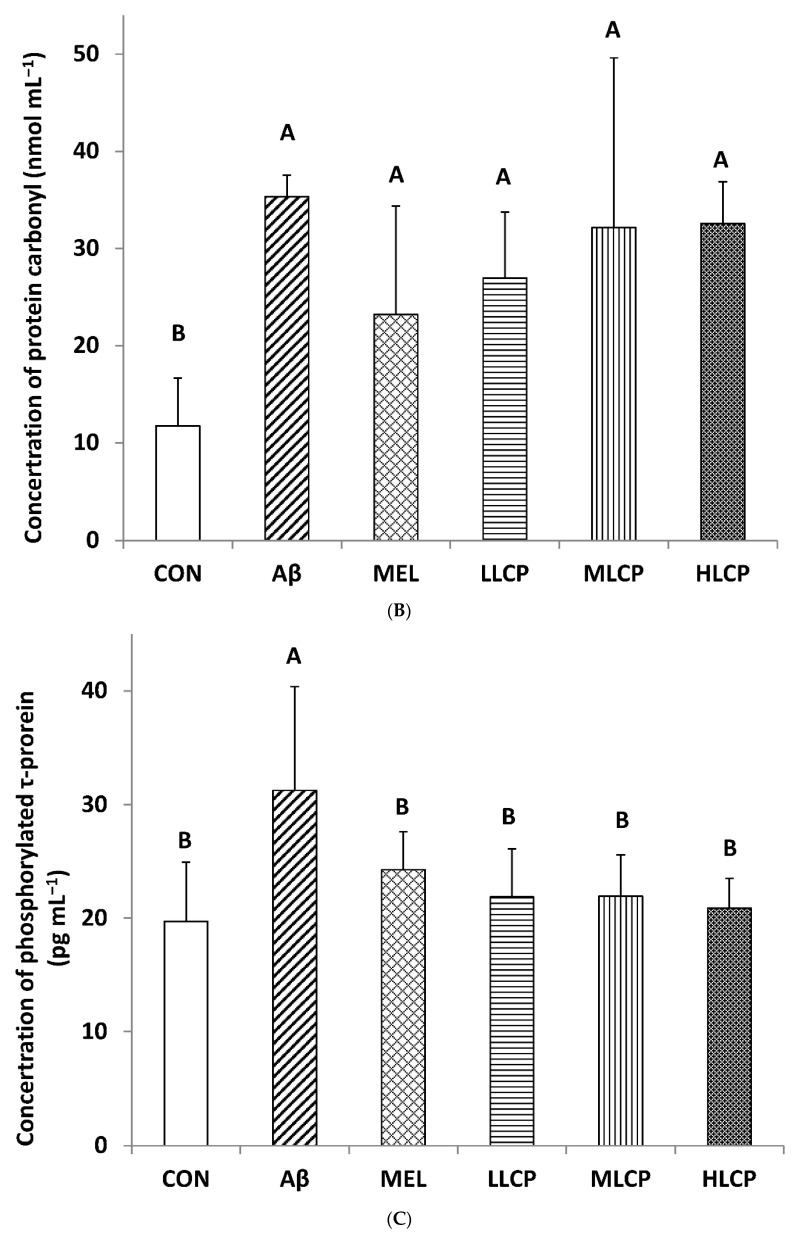

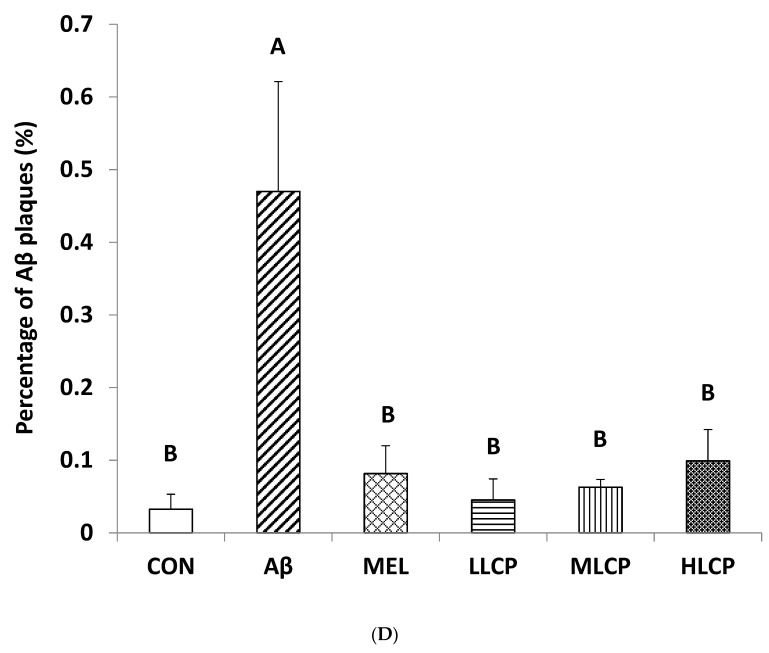
Effects of LCP fruit powder on oxidation stress and Alzheimer’s disease pathogenic factors using enzyme-linked the immunosorbent assay method in Aβ-induced Alzheimer’s mice brains. (**A**) The concentration of malondialdehyde (MDA). (**B**) The concentration of protein carbonyl. (**C**) The concentration of phosphorylated τ-protein. (**D**) The percentage of Aβ plaques. Values are expressed as mean ± SD (*n* = 8). Means with different letters (A and B) are significant differences between the groups (*p* < 0.05). Abbreviations: symbols are represented in Table 2.

MDA was the primary product of oxidative lipid degradation and a marker for oxidative stress in the tissue. The effects of feeding with LCP on lipid peroxidation MDA products in mice brains are shown in Figure 4A. Concentrations of MDA in five Aβ-injected treatment groups (Aβ, MEL, LLCP, MLCP, and HLCP) were significantly higher than that In the CON group (1.79 ± 3.70 µM, *p* < 0.05). Compared with these five groups, the level of MDA in the Aβ group (18.98 ± 4.38 µM) was significantly higher than the MEL, LLCP, MLCP, and HLCP groups (8.25 ± 3.24, 11.40 ± 1.71, 10.39 ± 2.01 and 8.77 ± 1.60 μM, respectively) (*p* < 0.05). Therefore, the results showed that feeding with MEL and LCP powder could significantly inhibit MDA production in mice brains.

The concentration of protein carbonyl in the Aβ group (35.34 ± 2.20 nmol/mL) was significantly higher than that in the CON group (11.74 ± 4.94 nmol/mL) in Figure 4B (*p* < 0.05). However, compared with these five Aβ-injected treatment groups, protein carbonyl contents in the MEL and LCP groups ranged from 23.22 to 32.58 nmol/mL and had no significant differences compared with the Aβ group (*p* < 0.05).

The results of phosphorylated τ-protein content in each group were shown in Figure 4C. The contents of Aβ_1–40_ -induced phosphorylated τ-proteins in the MEL, LLCP, MLCP, and HLCP groups were 21.70 ± 4.03, 22.81 ± 2.20, 23.45 ± 4.23, and 21.52 ± 2.90 pg/mL, respectively. They were significantly lower than that of the Aβ group (28.78 ± 9.06 pg/mL) (*p* < 0.05).

### 3.8. Effects on Aβ Plaque Accumulation in the Brain

Figure 4D shows the quantification data of Aβ plaque accumulation images. It was found that Aβ plaque accumulation in the Aβ group (0.46 ± 0.15%) was significantly higher than that in the CON group (0.03 ± 0.02%) (*p* < 0.05). Conversely, compared with the Aβ group, the values of MEL (0.08 ± 0.03%) and different dosages of LCP groups (0.04 ± 0.02, 0.06 ± 0.01, and 0.09 ± 0.04%, respectively) could significantly reduce the amounts of Aβ plaque.

Based on the above results, the biochemical parameters of each group, when orally administered with a medium and high dosage of LCP, showed that serum LDL-C decreased and the HDL-C/LDL-C ratio increased, verifying the hypolipidemic effect. Furthermore, the results of feeding with LCP fruit powder in Aβ-induced an Alzheimer’s mice model showed the inhibition of oxidative stress (including MDA and phosphorylated τ-protein) levels in the brain and prevented brain atrophy.

## 4. Discussion

### 4.1. Brain Tissue Weight and Serum Biochemical Parameters

Lessard-Beaudoin et al. demonstrated that the age-related weight, volume modifications of the whole brain and some cerebral regions decreased with age in different animal models. The total brain weight increased from 3 months to 12 months of age in C57BL/6J mice. With a normalized body weight, a decreasing trend was observed [34]. In Alzheimer’s disease patients, the excessive oxidative pressure in the brain caused a large amount of amyloid-like protein accumulation and τ-protein hyperphosphorylation. The oxidative stress also damaged the hippocampal gyrus and endothelial layer, promoting brain atrophy and reducing brain weight [35]. In addition, Figure S1A,B shows the results of H&E-stained sections of the brain tissue observed under a 40× and 400× microscope. The lateral brain injection of amyloid-like protein surgery showed no apparent traces of injection needle insertion, cell damage, infiltration, inflammation, or damage found in the brain tissue. Therefore, it could be inferred that the intracranial injection of amyloid-like protein caused cognitive impairment in Aβ mice. Therefore, the results of this experiment were consistent with the literature mentioned above, and orally administered mice with HLCP powder had no prominent atrophy. The serum lipid-related parameters in the HLCP group were significantly improved and showed that LCP powder could regulate the blood lipid parameters in AD mice.

### 4.2. Behavior Evaluation and Dose-Related Response

Many studies showed that mice’s memory and learning ability induced by lateral brain injection of amyloid-like protein was reduced, and the time to find the escape platform was rather lengthy [22,36]. Summarizing the results of the first and third days in our study, the reduction ratio of escape latencies for CON, MEL, LLCP, MLCP, and HLCP groups were 72, 73, 74, 69, and 55%, respectively. These data showed that the performance of MEL, LLCP, and MLCP groups meaningfully improved the learning and memory ability of mice.

The percentage of alternation in the Aβ group showed a lower rate of correct responses than the other groups during the T-maze discrimination task. This result is consistent with Liang et al.; that this response leads to a detriment in exploration ability, spatial learning, and memory in AD mice [25].

The plaque deposits in the brain of Alzheimer’s disease patients produced severe oxidative stress, which led to the increment of the lipid peroxidation product, malondialdehyde, in the brain, and caused damage to the brain nerve cells and memory decline [37]. Protein oxidation was also an essential factor associated with aging. The concentration of protein carbonyl commonly used was a hallmark of protein oxidation. Under high oxidative stress, the phosphorylated τ-protein would aggregate and cause the destabilization of microtubules in the brain of AD patients [9,38].

The scavenging activity of DPPH and the radical and inhibition effects of lipid oxidation products by methanol extract from LCP fruit were 90.57 ± 0.07% and 88.94 ± 0.27%, which showed that the methanol extract of LCP fruit had an intense antioxidant activity [13]. The administration of the 100~300 mg/kg LCP essential oil by ICR mice could significantly increase the frequency and duration of the oil remaining in the open arm, regulate the central nervous activity, and reduce anxiety in an elevated plus-maze test. The essential oil also had an analgesic effect and prolonged the sedation time of mice after anesthesia. Furthermore, it showed that the essential oil of LCP ameliorated depression [28]. The anti-dementia effects of limonene in lemon essential oil showed a strong ability to improved memory impaired by scopolamine [39]. The major components detected in the *Aloysia citrodora* essential oil were limonene, geranial, and neral, etc., respectively. These compounds demonstrated significant antioxidant, radical-scavenging and protective effects in β-amyloid-induced neurotoxicity [40]. The high levels of phenolic acids and flavonoid compounds further demonstrated the antioxidant, hemolytic lethality, and cytotoxicity activities of *L. cubeba* fruit extracts [41]. Based on the above findings, it could be postulated that LCP was utilized in dietary applications to reduce oxidative pressure and related damage in in vivo and in vitro studies.

**Figure 5:**
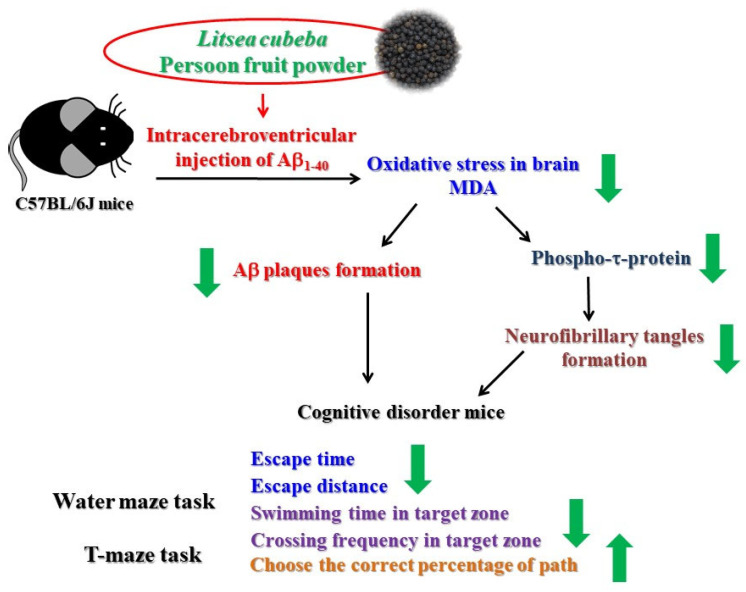
Hypothetical diagrams for roles of LCP fruit powder in Aβ-induced Alzheimer’s mice. LCP: *Listea cubeba* (Lour.) Persoon; Aβ: Amyloid β; MDA: Malondialdehyde; p-τ-protein: Phosphorylated tau protein; NFT: Neurofibrillary tangle.

Therefore, the feeding of MEL and three dosages of LCP powder resulted in a significant decrease in the escape time and distance by the water maze and T-maze task for learning and memory ability tests. LCP also increased the residence time and crossing frequency in the target zone of the water maze test and the probability of choosing the correct arm in the T-maze test than those from the Aβ group. However, there is no dose-effect between different groups in memory and learning tests, but the deposition of brain Aβ plaques and oxidative stress-related biochemical parameters (including the levels of MDA, phosphorylated τ-protein, and protein carbonyl) were increased in AD mice. Furthermore, it was found that this significantly retarded these changes in mice and repaired their memory ability when fed with a low dosage of LCP powder. The hypothetical diagrams for the roles of LCP fruit powder in Aβ-induced Alzheimer’s mice are summarized in Figure 5.

## 5. Conclusions

Feeding with the LCP powder could inhibit oxidative stress in the brain and prevent lipid peroxidation products. The levels of the Aβ plaque accumulated in the MEL and LCP groups’ brains were also significantly reduced. The low dosage of LCP powder in this animal model could ameliorate memory and learning ability in Aβ-induced Alzheimer’s disease mice. The recommended dosage of 30.2 mg per mouse per day converts to an equivalent dosage in humans of about 5.89 g of dried LCP powder per day. Therefore, LCP has excellent potential to develop a functional drug for the prophylaxis of AD.

## Figures and Tables

**Table 1:** Proximate compositions and essential oil content of *Litsea cubeba* Persoon fruit.

Composition	Content (%)
Moisture	65.26 ± 0.04
Crude ash	1.10 ± 0.08
Crude protein	4.37 ± 0.10
Crude fat	10.18 ± 0.27
Carbohydrate	19.07 ± 0.23
Essential oil recovery	3.80 ± 0.13

Values are expressed as mean ± SD (*n* = 3).

**Table 2:** Effects of *Listea cubeba* Persoon fruit powder on escape time and distance of the reference memory task in Aβ-induced Alzheimer’s mice.

Times Ordinal	CON	Aβ	MEL	LLCP	MLCP	HLCP
	Escape Latency (sec)					
First day	_a_ 76.46 ± 12.37 ^A^	_a_ 84.31 ± 10.66 ^A^	_a_ 77.09 ± 11.99 ^A^	_a_ 80.50 ± 14.32 ^A^	_a_ 80.25 ± 6.49 ^A^	_a_ 74.31 ± 14.54 ^A^
Second day	_b_ 30.31 ± 10.43 ^D^	_a_ 85.37 ± 6.95 ^A^	_b_ 48.40 ± 23.26 ^BC^	_b_ 37.40 ± 15.36 ^CD^	_b_ 49.93 ± 15.80 ^BC^	_b_ 56.40 ± 12.09 ^B^
Third day	_c_ 17.34 ± 10.07 ^C^	_a_ 77.56 ± 14.62 ^A^	_c_ 20.06 ± 9.85 ^C^	_c_ 21.25 ± 10.61 ^C^	_c_ 20.09 ± 7.70 ^C^	_c_ 33.62 ± 11.64 ^B^
	**Escape Distance (m)**					
First day	_a_ 9.73 ± 1.33 ^A^	_ab_ 10.33 ± 2.10 ^A^	_a_ 10.74 ± 1.57 ^A^	_a_ 10.30 ± 1.94 ^A^	_a_ 10.72 ± 1.43 ^A^	_a_ 9.39 ± 1.24 ^A^
Second day	_b_ 5.31 ± 1.45 ^C^	_a_ 10.71 ± 1.73 ^A^	_b_ 7.36 ± 3.21 ^BC^	_b_ 5.52 ± 1.66 ^C^	_b_ 08.55 ± 1.45 ^B^	_a_ 8.67 ± 1.95 ^B^
Third day	_c_ 3.00 ± 1.71 ^C^	_b_ 8.24 ± 2.29 ^A^	_c_ 2.90 ± 1.56 ^C^	_c_ 3.23 ± 1.65 ^C^	_c_ 3.52 ± 1.27 ^BC^	_b_ 5.07 ± 1.68 ^B^

Means with different superscript letters (A and B, A and C, B and C or A and BC….and so on) within a row indicate significant differences (*p* < 0.05). Means with varying letters of subscript (a and b, a and c or b and c) within a column indicate significant differences (*p* < 0.05). Values are expressed as mean ± SD (*n* = 8). CON: Control group; MEL: Melatonin group; Aβ: Amyloid β protein-induced group; LLCP: Low dosage of *Litsea cubeba* fruit powder; MLCP: Medium dosage of *Litsea cubeba* fruit powder; HLCP: High dosage of *Litsea cubeba* fruit powder.

## Data Availability

Not applicable.

## Supplementary Materials

The following are available online. Table S1. Effects of oral administration *Listea cubeba* Persoon fruits powder on body weight in Aβ-induced Alzheimer’s mice, Table S2. Effects of of *Listea cubeba* Persoon fruit powder on relative organ weight (%) in Aβ-induced Alzheimer’s mice, Table S3. Effects of *Listea cubeba* Persoon fruit powder on serum biochemical values in Aβ-induced Alzheimer’s mice.
