# Supplementary material for: Ameliorating Effect on Aβ-Induced Alzheimer’s Mice by Litsea cubeba Persoon Powder
Source: Molecules. 2021 Sep 21;26(18):5709. doi: 10.3390/molecules26185709 (PMC8469224; doi:10.3390/molecules26185709)
Supplement: Supplementary file 1 [file molecules-26-05709-s001.zip › molecules-1349787-supplementary.pdf]

Table S1. Effects of oral administration *Listea cubeba* Persoon fruits powder on body weight in Aβ-induced Alzheimer's mice.

Parameters/Group	CON	Aβ	MEL	LLCP	MLCP	HLCP
Initial body weight (g/mouse)	29.76 ± 1.87^{A}	28.31 ± 1.92^{A}	28.63 ± 1.96^{A}	28.90 ± 1.79^{A}	29.68 ± 1.81^{A}	28.22 ± 1.74^{A}
Body weight at week-8 (g/mouse)	31.28 ± 1.48^{A}	30.07 ± 2.15^{A}	30.05 ± 1.99^{AB}	30.32 ± 1.76^{AB}	29.62 ± 1.66^{AB}	28.72 ± 1.30^{B}

values are expressed as mean ± SD (n=8). Means with different superscript letters within a row indicate significant differences ($p< 0.05$). Means with the same superscript letters within a row are no significant difference between these values ($p< 0.05$). Abbreviations: symbols in-group represent in the Figure 2.

Table S2. Effects of of *Listea cubeba* Persoon fruit powder on relative organ weight (%) in Aβ-induced Alzheimer's mice.

Organ/Group	CON	Aβ	MEL	LLCP	MLCP	HLCP
Brain	1.49 ± 0.05^{B}	1.47 ± 0.10^{B}	1.54 ± 0.11^{AB}	1.53 ± 0.11^{AB}	1.55 ± 0.08^{AB}	1.61 ± 0.08^{A}
Heart	0.51 ± 0.03^{A}	0.51 ± 0.08^{A}	0.44 ± 0.06^{A}	0.45 ± 0.07^{A}	0.47 ± 0.05^{A}	0.46 ± 0.08^{A}
Liver	4.38 ± 0.33^{A}	4.41 ± 0.64^{A}	4.29 ± 0.31^{AB}	4.08 ± 0.08^{AB}	4.10 ± 0.09^{AB}	3.97 ± 0.25^{B}
Kidney	1.05 ± 0.07^{A}	1.07 ± 0.07^{A}	1.04 ± 0.05^{A}	1.03 ± 0.05^{A}	1.07 ± 0.06^{A}	1.06 ± 0.03^{A}

Relative organ weight = (organ weight/body weight) x 100%.

Values are expressed as mean ± SD (n=8). Means with different superscript letters within a row indicate significant differences ($p< 0.05$). Means with the same superscript letters within a row are no significant difference between these values ($p< 0.05$). Abbreviations: symbols represent in the Figure 2.

Table S3. Effects of *Listea cubeba* Persoon fruit powder on serum biochemical values in Aβ-induced Alzheimer's mice.

**Serum parameters/Group**	**CON**	**Aβ**	**MEL**	**LLCP**	**MLCP**	**HLCP**
AST (U/L)	43.12 ± 3.09^{A}	38.00 ± 3.74^{BC}	41.12 ± 4.29^{AB}	40.50 ± 3.38^{ABC}	40.12 ± 4.05^{ABC}	36.62 ± 2.92^{C}
ALT (U/L)	33.50 ± 4.98^{A}	28.50 ± 5.42^{BC}	29.50 ± 2.87^{B}	27.12 ± 2.58^{BC}	25.62 ± 3.99^{BC}	24.62 ± 2.61^{C}
BUN (mg/dL)	37.62 ± 1.77^{A}	34.56 ± 2.63^{BC}	33.67 ± 1.93^{C}	36.15 ± 2.52^{AB}	31.20 ± 1.58^{D}	27.80 ± 2.36^{E}
Creatinine (mg/dL)	0.31 ± 0.04^{A}	0.31 ± 0.03^{A}	0.29 ± 0.02^{A}	0.30 ± 0.02^{A}	0.31 ± 0.01^{A}	0.25 ± 0.03^{B}
Uric acid (mg/dL)	3.63 ± 0.67^{AB}	4.02 ± 1.78^{AB}	3.55 ± 1.02^{AB}	3.83 ± 0.74^{AB}	4.70 ± 1.06^{A}	3.18 ± 0.40^{B}
Cholesterol-total (mg/dL)	124.37 ± 9.73^{A}	124.75 ± 12.25^{A}	123.50 ± 13.45^{A}	119.12 ± 9.20^{AB}	117.25 ± 9.43^{AB}	108.25 ± 6.06^{B}
HDL-C (mg/dL)	92.50 ± 8.08^{A}	92.00 ± 11.32^{A}	92.12 ± 11.36^{A}	88.87 ± 7.64^{AB}	91.25 ± 6.88^{AB}	82.25 ± 4.26^{B}
LDL-C (mg/dL)	21.37 ± 2.55^{A}	19.75 ± 3.24^{AB}	18.25 ± 2.37^{AB}	17.87 ± 3.75^{BC}	16.75 ± 3.19^{BC}	14.75 ± 2.60^{C}
HDL-C/LDL-C	4.35 ± 0.43^{B}	4.77 ± 1.04^{AB}	4.95 ± 0.39^{AB}	5.15 ± 1.13^{AB}	5.59 ± 1.01^{A}	5.71 ± 0.99^{A}

Values are expressed as mean ± SD (n=8). Means with different superscript letters (A and B, A and C, A and D, A and E, B and C, B and D, B and E or A and BC….and so on) within a row indicate significant differences ($p< 0.05$). Means with the same superscript letters (A and AB, AB and B, BC and C or ABC and A, B, or C…. and so on) within a row are no significant difference between these values ($p< 0.05$). AST: aspartate aminotransferase; ALT: alanine aminotransferase; BUN: blood urea nitrogen. Abbreviations: symbols in-group represent in the Figure 2. Effects on escape time of reference memory task

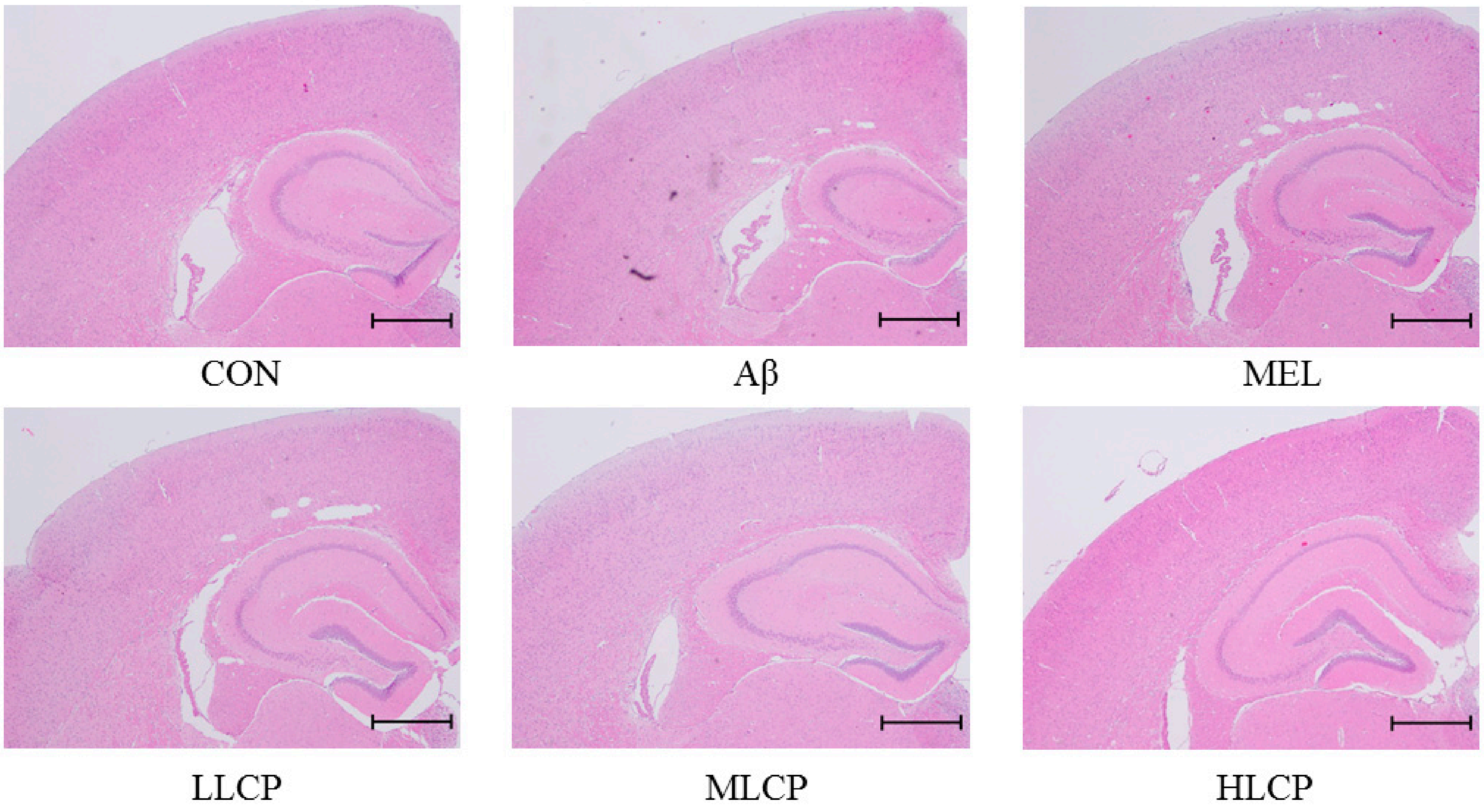

Figure S1(A) Effects of *Listea cubeba* Persoon fruit powder on histopathological alterations of brain in Aβ-induced Alzheimer's mice. The images were taken 40X magnification with H&E staining. Scale bars = 600 μm.

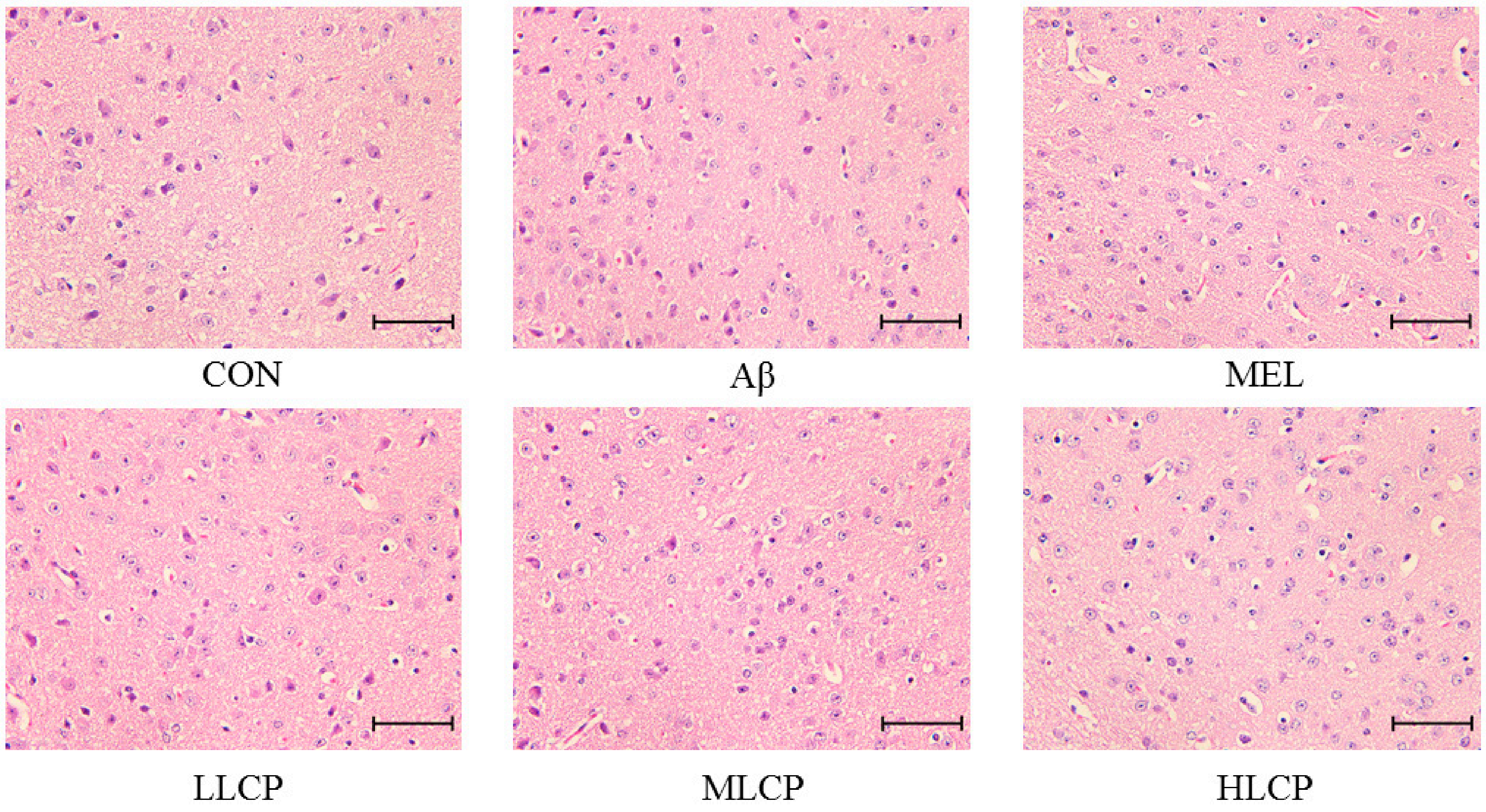

Figure S1(B) Effects of *Listea cubeba* Persoon fruit powder on histopathological alterations of brain in Aβ-induced Alzheimer's mice. The images were taken 400X magnification with H&E staining. Scale bars = 60 μm.
